# Influence of geographic isolation and the environment on gene flow among phenotypically diverse lizards

**DOI:** 10.1038/s41437-024-00716-y

**Published:** 2024-09-12

**Authors:** Thomas J. McGreevy, Nicholas G. Crawford, Pierre Legreneur, Christopher J. Schneider

**Affiliations:** 1 Department of Biology, Boston University, 5 Cummington Mall, Boston, MA 02215 USA; 2 Department of Natural Resources Science, University of Rhode Island, 1 Greenhouse Road, Kingston, RI 02881 USA; 3 CRIS EA 647 (P3M), University of Lyon, Villeurbanne, France

**Keywords:** Ecological genetics, Genetic variation, Adaptive radiation

## Abstract

Lizards in the genus *Anolis* comprise hundreds of species that display a wide range of phenotypic variation closely related to their environment. One example is the Guadeloupean anole (*Anolis marmoratus* ssp.) that display extreme phenotypic variation, primarily in adult male color and pattern, with twelve described subspecies on the archipelago. Here we examine the relationship between phenotypic and genetic divergence among five subspecies on the two main islands and test the role of geographic isolation and the environment in reducing gene flow. We also examined two offshore island populations to assess the impact of complete geographic isolation on gene flow. We analyzed color phenotypes by measuring spectral reflectance and genomic diversity using SNPs. Genetic divergence was correlated with dorsolateral head and body color phenotypes, and slope and geographic distance were nearly equivalent at explaining this divergence. There was minimal genome-wide divergence at neutral loci among phenotypically disparate subspecies on the two main islands and their differentiation is consistent with a model of divergence with gene flow. Our spatial visualization of gene flow showed an impact of environmental features consistent with a hypothesis of ecologically driven divergence. Nonetheless, subspecies on the two main islands remain interconnected by substantial gene flow and their phenotypic variation is likely maintained at selection-gene flow equilibrium by divergent selection at loci associated with their color phenotypes. Greater isolation, such as inhabiting a remote island, may be required for reducing gene flow. Our findings highlight the role of the environment, adaptation, and geographic isolation on gene flow.

## Introduction

Identifying the factors involved in influencing gene flow among populations and their pattern of genetic diversity is critical for understanding evolutionary processes. Gene flow can be limited due to the inability of animals to interact and interbreed and can create a pattern of isolation-by-distance (IBD) where genetic divergence is correlated with geographic distance (Wright 1943). The genetic divergence can be further increased by genetic drift in the isolated populations. Gene flow also can be influenced by abiotic (e.g., elevation, slope, and precipitation) and biotic (e.g., vegetation) factors and create a pattern of isolation-by-environment (IBE; Wang and Summers 2010; Reviewed in Wang and Bradburd 2014). The degree to which IBD and IBE influence a population’s gene flow and pattern of genetic diversity has previously been investigated (Bradburd et al. 2013; Shafer and Wolf 2013; Wang et al. 2013; Sexton et al. 2014), but studies that directly analyze the environmental factors that impact gene flow are scarce and further complicated by the fact that IBD and IBE can influence a system simultaneously. A third model that is related to IBE is isolation-by-adaptation (IBA) where genetic divergence is predicted to correlate with morphological divergence (Nosil et al. 2008). To test among these models, multiple comparisons among phenotypically diverse populations are needed to make stronger conclusions and underlying population structure could confound analyses if they have different demographic histories. Thus, an ideal animal system is one with a broad range of morphological variation over a limited geographic scale that has a diverse range of environmental variation, but limited population structure.

Lizards in the genus *Anolis* are one of the best-studied vertebrate adaptive radiations with repeated, independent evolution of ecomorphs across multiple islands (Losos 2009). Large islands are thought to provide both a degree of allopatry (i.e., IBD or historical isolation) as well as a diversity of habitats that would promote divergent natural selection and speciation. By contrast, microevolutionary analyses of population variation and local adaptation on smaller islands suggest that morphological divergence occurs in response to natural selection over small geographic scales (e.g., on the order of a few kilometers) and that the process of divergence may occur in parapatry (Malhotra and Thorpe 1991; Thorpe et al. 2005). Support for this assertion comes from studies demonstrating that intraspecific variation is strongly associated with habitat or environmental variation (i.e., suggesting local adaptation) that, in some instances, results in reduced gene flow among populations (Ogden and Thorpe 2002; Stenson et al. 2002).

In our study system, the Guadeloupean anole (*Anolis marmoratus* ssp.) subspecies complex are found on the Guadeloupe archipelago that consist of two main islands (Basse Terre and Grande Terre), which are considered small islands for anole speciation (Losos and Schluter 2000). The geographic setting of diversification in the Guadeloupean anole complex is important because of the contrasting complexity between the two main islands. Basse Terre is a complex volcanic island, dominated by a central, north-south volcanic chain varying in age and elevation from older and lower in the north (with an estimated age of 1.5–3 MYA; reviewed in Samper et al. 2007), to younger and higher in the south, capped by the still active La Souffriere volcano (elev. 1447 m), which has erupted 16 times during the Holocene (Global Volcanism Program 2024). The volcanic mountain chain intercepts the easterly trade winds resulting in heavy rainfall on the eastern side of the island, with a distinct rain shadow to the west. Variation in elevation and rainfall results in habitat gradients, both longitudinally and altitudinally, over short geographic distances. These gradients vary from strong (e.g., from closed, wet tropical forest to dry thorn scrub over an east-west, straight line distance of only 10–15 km in the south of Basse Terre) to weaker, more gradual transitions in the northern part of the island where the mountain chain is lower and concomitant rain shadow effect is lesser. Also, there are shallower rainfall gradients along both coasts of Basse Terre, with the eastern lowlands being very wet in the south and mesic in the north, whereas the west coast displays the opposite gradient with mesic conditions in the north and xeric habitats in the south. In contrast to Basse Terre, Grande Terre is a relatively flat, limestone-capped island with a volcanic base (Lazell 1964, 1972; Samper et al. 2007). The limestone cap of Grande Terre is estimated to be ca. 0.4 MY old, suggesting that the island’s last submergence was in the mid- to late-Pleistocene. Grande Terre is slightly uplifted in the southwest and slopes gently to the east, with a more abrupt transition from upland to lowland along a north-south axis. The proximity of Grande Terre to Basse Terre results in a rainfall gradient with higher rainfall and mesic habitats in the southwest and decreasing rainfall and more xeric habitats to the east and north of the island. Finally, there are several other islands in the Guadeloupean archipelago that vary from small islands of volcanic origin (e.g., Ilets Pigeon) to moderately large, limestone capped islands (e.g., Marie Galante). The habitats on these offshore islands do not vary greatly from adjacent habitats on the mainland.

Guadeloupean anole display striking variation in phenotype, which led Lazell (1964) to describe 12 subspecies within the complex. Phenotypic variation on the two main islands, which are inhabited by six subspecies, is associated with habitat and likely influenced by both ecological and sexual selection (Muñoz et al. 2013). Muñoz et al. (2013) combined microsatellite genetic data with body coloration data among Guadeloupean anoles spanning the entirety of Grande Terre and found strong phenotypic divergence despite high gene flow among sites, suggesting a role for natural selection in structuring phenotypic variation across the landscape. This association between habitat and body color is presumably adaptive as it is consistent with a larger, macroevolutionary correlation between habitat and body color among species of *Anolis* (Losos 2009). For example, the Grand Cayman anole (*A. conspersus*) shows high variation in body color with green body colored individuals inhabiting areas with the highest precipitation and brown body colored individuals inhabiting areas with the lowest precipitation (Macedonia 2001). A recent comprehensive comparison of six Lesser Antilles anole species found the majority of species showed a pattern of parallelism in their dorsal coloration that is related to precipitation (Yuan et al. 2023).

In our study, we expanded upon the work of Muñoz et al. (2013) by comparing Guadeloupean anole samples from Grande Terre with those from the more environmentally-diverse island of Basse Terre. We used a landscape genetic approach to: (1) characterize the population genetic structure and phenotypic color variation of the Guadeloupean anoles and (2) determine the relative contribution of IBD, IBA, and IBE in reducing gene flow in our system. We also examined the impact of geographic isolation on genetic divergence among subspecies via comparison of the two main islands to a near-shore island population (Ilets Pigeon) and a more distant island population of Marie-Galante anoles (*A. ferreus*). Although the Marie-Galante anole is currently considered a distinct species (Gorman and Kim 1976), it was formerly classified as a subspecies of Guadeloupean anole (Lazell 1964;1972), and its taxonomic relationships with Guadeloupean anoles is not fully resolved (Schneider et al. 2001). Nuclear genetic analyses have not been done to confirm their designation as a species. However, we use Marie-Galante anole to represent the furthest end of the speciation spectrum in Guadeloupean anoles. We hypothesized that subspecies from type localities would form distinct genetic clusters according to Lazell (1964). We also hypothesized environmental variables would be more important than IBD at reducing gene flow and these environmental differences would have a stronger role in reducing gene flow on the more environmentally diverse Basse Terre compared to Grande Terre.

## Materials and methods

### Study species

**Fig. 1:**
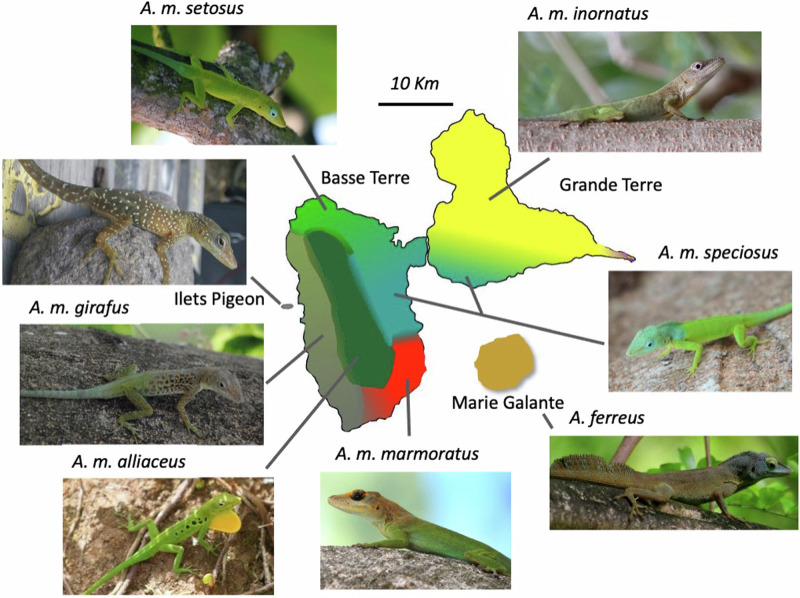
Subspecies transition zones on Guadeloupe. Guadeloupean anole (*Anolis marmoratus* ssp.) subspecies complex on the two main islands of Guadeloupe, Basse Terre and Grande Terre. Shaded areas show the relative distribution of each subspecies and was adapted from Muñoz et al. (2013). Samples also were collected on two offshore islands, Ilets Pigeon and Marie Galante.

Within *A. marmoratus*, six subspecies are found on the two main islands and five are confined to smaller, offshore islands on the same island bank (Lazell 1964, 1972). Phenotypic variation among subspecies on the two main islands is primarily in color and pattern of adult males (Fig. 1; Lazell 1964, 1972). Basse Terre contains five subspecies that are distributed continuously throughout the island and show geographically structured clinal variation in phenotype (Lazell 1972). On the immediately adjacent island of Grande Terre, there are two subspecies, one of which is shared with Basse Terre. The two subspecies on Grande Terre are continuously distributed and show clinal variation across the habitat gradients from the mesic to the xeric portions of the island. The subspecies (*A. m. inornatus* and *A. m. girafus*) found in dry, open habitats have a generally cryptic, dull brown-green body and head color, whereas the other subspecies (*A. m. marmoratus*, *A. m. setosus*, *A. m. speciousus*), occupying more mesic to wet habitats, have a bright green body and conspicuous head color. The sixth subspecies, *A. m. alliaceus*, has a bright green body with dark spots and occupies the central highlands on Basse Terre in canopy that can be 40 meters high (Lazell 1964).

### Sample collection and DNA extraction

**Fig. 2:**
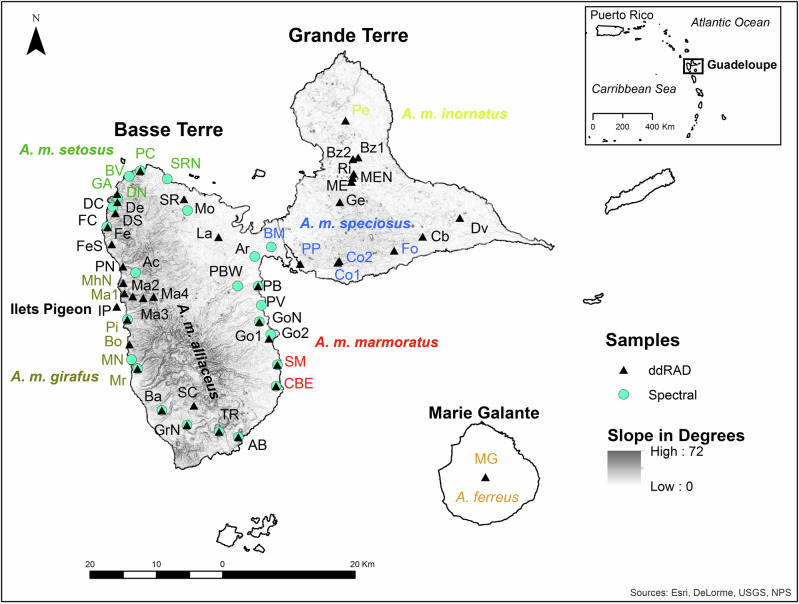
Genetic and phenotypic sampling sites. Map of the study area showing the approximate distribution and sample locations of Guadeloupean anole (*Anolis marmoratus* ssp.) on the two main islands of Guadeloupe (Basse Terre and Grande Terre) and the offshore island of Ilets Pigeon. The Marie-Galante anole (*A. ferreus*) samples were from the island of Marie Galante. Site abbreviations are listed in Table 1. The sites that are the same color of the subspecies name are characteristic of that particular subspecies according to Lazell (1964). Slope is only shown for Basse Terre and Grande Terre.

We collected 280 tissue samples from the tail tips of live caught anoles from 39 sites on the two main Guadeloupe islands (Basse Terre *n* = 192 and Grande Terre *n* = 72) and two offshore islands (Ilets Pigeon *n* = 8 and Marie Galante *n* = 8) from 1988 to 2012 (Table 1; Fig. 2). We were not able to collect type samples from *A. m. alliaceus* because we were unable to obtain a permit to collect lizards from the National Park. Most anoles caught were adult males (*n* = 271), but we also included seven juvenile males and two adult females in our genetic analyses. We targeted the analysis of males because they display diverse phenotypes. The average number of samples per site was seven with a range of two to 11. The tissue samples were stored in >90% ethanol and transported at room temperature to the United States where they were stored at −20 °C. We extracted genomic DNA from the tissue samples using the Qiagen™ DNAEasy kit (Carlsbad, CA) according to manufacturer’s instructions.

**Table 1:** Sample locality names for the areas where the double digest Restriction-site Associated DNA (ddRAD) and spectral analysis samples originated from.

Locality	Site	X (UTM)	Y (UTM)	ddRAD (n)	Phenotype (n)	Both (n)
Anse Bernard	AB	647744.37	1768246.66	9	19,10,10,10,10^+^	9
Acomat	Ac	632247.35	1793045.69	0	20,10,10,10,10	0
Arnouville	Ar	650232.09	1795487.33	0	2,0,2,2,2	0
Baillif	Ba	636203.74	1772267.89	9	20,10,10,10,10^+^	9
Baie Mahault^2^	BM	652706	1796937	0	4,0,4,4,4	0
Bouillante^3^	Bo	631321.31	1782235.83	6	0	0
Bas Vent^4^	BV	631264.97	1807613.67	0	20,11,10,10,10	0
Bazin	Bz1	665851.23	1810458.24	4	5*^+^	0
Bazin	Bz2	665039.95	1810232.95	1	0	0
Chateaubrun	Cb	675577.57	1798511.14	8	5*^+^	0
Capesterre-Belle Eau^1^	CBE	653433.01	1775918.35	7	35,9,26,26,27^+^	7
Cocoyer01^2^	Co1	662769.01	1794583.31	4	0	0
Cocoyer02^2^	Co2	662991.69	1794828.41	4	5,0,5,5,5*^+^	0
Deshaies	De	629484.61	1803723.82	2	0	0
Deshaies Coast	DC	628800.05	1802978.5	0	20,10,10,10,10^+^	0
Deshaies North^4^	DN	629510	1804341.4	0	20,10,10,10,10^+^	0
Deshaies South	DS	629186.41	1802036.17	6	0	0
Desvarieux	Dv	681181.2	1801337.9	9	10*^+^	0
Ferry	Fe	628027.85	1799949.32	10	0	0
Ferry Coast	FC	627893.21	1800005.75	0	20,10,10,10,10^+^	0
Ferry South	FeS	628636.48	1797365.16	8	0	0
Fouche^2^	Fo	671275.67	1796385.53	9	9*^+^	0
Grande Anse^4^	GA	629457.04	1804945.86	4	0	0
Gensolin South	Ge	663065.35	1803722.44	8	0	0
Goyave	Go1	652361.05	1783111.45	7	0	0
Goyave	Go2	652649.91	1783719.81	0	18,9,9,9,9^+^	0
Goyave North	GoN	650920.74	1785634.36	6	9,0,9,9,9	6
Gourbeyre North	GrN	639991.69	1770063.09	9	20,10,10,10,10^+^	9
Ilets Pigeon	IP	629392	1787923	8	0	0
Lamentin	La	644748.07	1798429.17	10	0	0
Mahaut^3^	Ma1	630548.97	1789948.09	4	0	0
Mahaut	Ma2	631804.56	1789489.67	4	0	0
Mahaut	Ma3	633376.85	1789235.66	7	0	0
Mahaut	Ma4	634967.43	1789421.14	8	0	0
Morne-A-l’Eau	ME	664825.83	1806771.03	4	0	0
Morne-A-l’Eau North	MEN	664992.17	1807380.91	3	4*^+^	0
Marie Galante	MG	685067.19	1762213.35	8	0	0
Mahaut North^3^	MhN	630363.73	1791517.78	5	0	0
Morne Rouge	Mo	640078	1802439	0	16,8,8,8,8	0
Marigot^3^	Mr	632524.74	1778566.54	8	13,7,7,7,7^+^	7
Marigot North^3^	MN	631600.39	1779939.77	0	4,4,4,4,4	0
Petit Bourg	PB	650744.12	1791022.9	7	10,0,10,10,10^+^	7
Petit Bourg West	PBW	647629.16	1791051.77	0	6,0,6,6,6	0
Plage de Clugny^4^	PC	632944.6	1808444.86	11	23,8,15,15,15^+^	7
Pelletan^5^	Pe	663937.44	1816023.1	6	6*^+^	0
Pigeon^3^	Pi	630998.51	1785964.54	7	22,0,22,22,22^+^	7
Pointe Noire	PN	630322.78	1793995.75	6	0	0
Pointe-a-Pitre^2^	PP	657088.28	1794398.88	8	28,8,27,28,28*^+^	0
Plage de Viard	PV	651212.63	1788144.07	6	5,0,5,5,5^+^	0
Richeval	Ri	665154.08	1808008.47	4	0	0
St. Claude	SC	641033.36	1772988.97	6	0	0
Saint Marie^1^	SM	653644.15	1779149.65	7	26,0,26,26,26^+^	7
Saint Rose	SR	639544.66	1804150.17	4	0	0
Saint Rose North^4^	SRN	637005.39	1807215.36	0	16,8,8,8,8^+^	0
Trois Riveires North	TR	644841.85	1769058.52	9	18,9,9,9,9^+^	8

Location coordinates are in Universal Transverse Mercator (UTM). The Phenotype column shows the spectral analysis sample size (n) for dorsolateral head, eye ring, dewlap, dorsolateral body, and lateral tail. The sites that have phenotypes characteristic of only one subspecies are designated as 1 – *Anolis marmoratus marmoratus*, 2 – *A. m. specious*, 3 – *A. m. girafus*, 4 – *A. m. setosus*, and 5 – *A. m. inornatus* according to Lazell (1964). The Both column shows the sample size for ddRAD and spectral analyses that were conducted on the same samples. Phenotype sample sizes for Grande Terre from Muñoz et al. (2013) are shown with an * and samples used for integrated genetic, environmental, and phenotypic analyses are denoted with a ^+^. Phenotype sample size that only have one value had the same value for all body parts.

### ddRADseq library preparation and single nucleotide polymorphism (SNP) calling

We used the double-digest Restriction-Site Associated DNA sequencing (ddRADseq) method of Peterson et al. (2012) to create sequencing libraries (Appendix S1). In brief, genomic DNA was standardized to 500 ng and digested with SphI-HF® (New England Biolabs, Ipswich, MA) and MluCI® (New England Biolabs, Ipswich, MA) according to manufacturer’s instructions. We size selected for 202 base pairs using 2% ethidium free cassettes on the Pippin Prep (Sage Science, Beverly, MA). The libraries were submitted to the Northwest Laboratory Bauer Core Facility at Harvard University for single-end 50 bp read sequencing using an Illumina HiSeq™ 2000 sequencing system (San Diego, CA). Six sets of libraries that each included approximately 48 samples were created. A total of eleven next-generation sequencing runs were conducted because of poor clustering on the flow cell. Four libraries were run one time, one library was run two times, and one library was run five times, which included one paired-end run. The Bauer Core Facility staff demultiplexed the raw sequencing data by each index. Boston University’s Scientific Computation and Visualization Cluster and the University of Rhode Island’s Bluewaves high performance computing were used to run all software programs, except for those run in RStudio version 1.2.5033 (Posit team 2019). The raw data was processed in two main batches. The first batch included all 280 samples, which included *A. ferreus* samples from Marie Galante. The second batch included all samples except the *A. ferreus* samples (*n* = 272).

The bioinformatics software Stacks version 2.41 (Catchen et al. 2011, 2013) was used to demultiplex the barcodes and quality control filter the reads. The *process_radtags* program was run to clean the data, discard reads with low quality scores, and rescue barcodes and RAD-Tags. Samples that were sequenced multiple times had their repeated sample files merged after the *process_radtags* step. The assembly parameters for the de novo processing of the samples were optimized by analyzing a subset of samples, two per site, and varying the parameters *M* (“number of mismatches allowed between the two alleles of a heterozygote sample”) and *n* (“number of mismatches allowed between any two alleles of the population”) to optimize the number of SNP loci (Rochette and Catchen 2017). The Stacks *populations* script was run with the following parameters: 28 as the minimum number of sampling locations a SNP has to be present in, 0.8 as the minimum percentage of individuals that have a SNP, 0.01 as the minimum minor allele frequency, three as the minimum minor allele count, and 0.7 as the maximum observed heterozygosity. The *write-random-snp* script in the *populations* program was used to randomly retain only one SNP per read. The program VCFtools version 0.1.16 (Danecek et al. 2011) was used to remove samples that had > 50% missing data. The R packages *SNPrelate* (Zheng et al. 2012) was used to estimate the relatedness of all samples to ensure no two samples were identical (>99% identical) and *vcfr* (Knaus and Grunwald 2016) was used to visualize the allele balance of each sample. Each SNP was tested for departure from Hardy-Weinberg equilibrium (HWE) using the script *filter_hwe_by_pop.pl* (https://github.com/jpuritz/dDocent/raw/master/scripts/) with a p-value cut-off of 0.01 (-h) and loci only having to depart from HWE in one sampling location (-c of either 0.03 or 0.06).

### Population genetics and isolation by distance

The *population* program in Stacks was used to estimate expected heterozygosity (H_e_), the inbreeding coefficient (F_IS_), and pairwise F_ST_ values. We tested for isolation by distance by conducting Mantel tests between the F_ST_/(1- F_ST_) and log base 10 of pairwise straight line distance values (Rousset 1997). Mantel tests were conducted for all sites (*n* = 36) as well as subsets of samples from either Basse Terre (*n* = 27) or Grande Terre (*n* = 9) using GenAlEx version 6.5 (Peakall and Smouse 2006, 2012) with 9999 permutations and an alpha level of 0.05 to determine the significance of the simulated *p* values. We also split Basse Terre sites in half at Pigeon (Pi) and Petit Bourg (PB) with those sites south (*n* = 13) and the remaining sites north (*n* = 14) to make the distance amongst the furthest sites more comparable to Grande Terre. We estimated the number of migrants per generation (Nm) using a two-dimensional stepping-stone model (Kimura and Weiss 1964; Hedrick 2011) for all sites on Grande Terre and Basse Terre.

### Population structure

We used non-spatial and spatially explicit analyses to test for population genetic structure. We conducted a non-spatial Principal Component Analysis (PCA) using the R package *adegenet* version 2.1.2 (Jombart 2008). To assess the impact of habitat variation on effective migration (gene flow) while accounting for the effects of isolation-by-distance, we used the spatially explicit analysis package Estimate Effective Migration Surfaces (EEMS; Petkova et al. 2016), which estimates effective migration based on a stepping-stone model (Kimura and Weiss 1964). This method identifies geographic areas where estimates of gene flow depart from expectations of isolation by distance and where the gene flow is potentially being influenced by environmental variables (Petkova et al. 2016). The difference matrix was estimated using the script *bed2diffs_v1*, which imputes missing SNP data and estimates the average pairwise genetic difference among all individuals. The proposal variances were optimized for an acceptance rate between 20 to 30% as recommended in the EEMS instruction manual (https://github.com/dipetkov/eems/blob/master/Documentation/EEMS-doc.pdf). The proposal variance acceptance rates are the percent of times the variance values for the proposal distributions for the effective migration and effective diversity parameters are accepted. We conducted three independent runs with different deme sizes (200, 400, 800, and 1000), averaged the runs, and displayed the results using R scripts described in the EEMS manual (https://github.com/dipetkov/eems/blob/master/Documentation/EEMS-doc.pdf). The effective migration rates are visualized on the log scale that is relative to the overall migration rate in a given area.

An additional spatially explicit method, MEMGENE (Galpern et al. 2014), was used to detect fine-scale population structure. MEMGENE is a regression-based technique that identifies spatial neighborhoods (i.e., groups of samples) in genetic data that may have been influenced by environmental variables (Galpern et al. 2014). MEMGENE is similar to spatial autocorrelation PCA analysis and is intended to identify fine-scale spatial genetic variation in cases where that variation may be subtle and where there is substantial gene flow among populations (Galpern et al. 2014). We used the same genetic difference matrix as our EEMS analysis along with the sample’s location to produce a Moran’s eigenvector map using the R package *MEMGENE*.

### Phenotypic analyses

We analyzed variation in adult male color and pattern as described by Muñoz et al. (2013). In short, we measured coloration of adult male *A*. *marmoratus* ssp. from Basse Terre at 24 locations, which included eight sites analyzed by Muñoz et al. (2013) (Table 1). We measured the reflectance of five body parts: dorsolateral head (temporal region), eye ring, dewlap (center), dorsolateral body, and lateral tail using an Ocean Optics USB 2000 field portable spectrometer (Dunedin, FL). The number of samples per site ranged from 2 to 35 with an average of 11 ± 7 individuals per site. We quantified variation in coloration for each body part using hue angle using the four-segment classification scheme of Endler (1990) to be able to combine our results with our previous work described in Muñoz et al. (2013). Hue angle was calculated using the python script spec.py (http://github.com/ngcrawford/Coloration).

To determine if phenotypic divergence was associated with genetic divergence, we used Mantel tests to estimate the correlation between a measure of genetic distance (pairwise F_ST_/(1- F_ST_)) and log base 10 transformed hue values. The pairwise differences in hue values were calculated by taking the absolute value difference between the average hue values of each body part at all sites. The Mantel tests included genetic and phenotypic data from either the same site or sites within close proximity, but we did not include all of our sites with phenotype data because they lacked genetic data near a given site. We also conducted the analyses separately for Basse Terre (n = 16 sites) and Grand Terre (n = 8 sites).

### Modeling environmental impact on gene flow

We used a Random Forest approach (Breiman 2001) and the R package *randomForest* (Liaw and Wiener 2002) to explicitly test the effect of environmental variables derived from remote sensing platforms on gene flow. The Random Forest method uses a regression-based machine learning algorithm that can simultaneously analyze correlated environmental variables. The method also has internal estimates of error, measures of variable importance (Breiman 2001), and can include complex interactions among independent variables (De’ath and Fabricius 2000; Murphy et al. 2010). The proportion of shared alleles (Dps) were estimated for samples from Basse Terre (*n* = 171) and Grande Terre (*n* = 54) separately using the R package *propShared* in *adegenet*. Seven environmental variables at 30-m resolution were included as explanatory variables, which included geographic and biological variables. The geographic variables included elevation (using a digital elevation model (DEM)), aspect, and slope, all derived from the Advanced Spaceborne Thermal Emission and Reflection Radiometer (https://asterweb.jpl.nasa.gov/gdem.asp). The biological variables included: Normalized Difference Vegetation Index (NDVI) from Landsat8 obtained from ESRI online (https://www.esri.com/en-us/home) and Tassled Cap (Kauth and Thomas 1976) estimates of brightness, greenness, and wetness based on the analysis of a Landsat7 ETM+ image (LE70010491999303EDC00) taken on October 30, 1999 (http://eros.usgs.gov). The biological variables were processed in ArcMap 10.5.1 (ESRI, CA). Brightness is a measure of soil reflectance, greenness is a measure of vegetation density, and wetness is a measure of soil and plant moisture (Crist and Cicone 1984). The model the Random Forest approach was trying to fit was Dps ~ DEM + aspect + slope + NDVI + Tassled Cap brightness + Tassled Cap greenness + Tassled Cap wetness.

We also conducted a Generalized Dissimilarity Modeling (GDM; Ferrier et al., 2007) analysis using the R package *gdm* 1.5.0–1 (Fitzpatrick et al. 2021). The GDM method is a matrix regression technique that can identify the variables influencing beta diversity or compositional turnover (Ferrier et al. 2007). The method uses I-splines to fit nonlinear relationships between response and predictor variables and has been used to determine the influence of environmental variables and geographic distance on genetic and morphological differentiation in lizards and birds (Thomassen et al. 2010; Kaliontzopoulou et al. 2018). We used pairwise F_ST_ values as the response variable for the 24 sites on Basse Terre and Grande Terre that had genetic and morphological data because F_ST_ is distance based, which is required by the GDM method. Our predictor variables were spectral reflectance hue values, bioclimatic variables Bio01 (annual mean temperature) and Bio12 (annual precipitation) at a 1-km spatial resolution from the WorldClim database (Hijmans et al. 2005), DEM, aspect, slope, and geographic distance. The default GDM settings were used by following the fitzlab tutorial (https://github.com/fitzLab-AL/gdm#section-2---advanced-spatial-analyses-using-gdm).

## Results

### SNP quality control filtering and calling

The eleven 50 bp HiSeq runs produced 1,024,064,486 reads. The Stacks *process_radtags* program retained 969,361,997 reads or 95% of the original raw reads. Samples with less than 100,000 reads were removed from analyses (*n* = 11). The average number of reads per sample was 3,522,075 (123,576 to 13,759,901) ± 2,452,331 standard deviation (SD). The average coverage was 21.8 ± 11.0 SD. The optimal Stacks parameters for all samples was one for both *M* and *n*. The Stacks filtering parameters retained 36,887 variable sites. Filtering samples with more than 50% missing data retained 240 samples. The test for departure from Hardy-Weinberg equilibrium identified 30 outlier SNPs, which were removed using VCFtools. The data set of all biallelic SNPs per locus retained 36,059 SNPs.

The optimal parameters for samples excluding *A. ferreus* was two for both *M* and *n*. The Stacks’ filtering parameters retained 36,158 biallelic SNPs and 232 samples when samples with more than 50% missing data were removed. The test for departure from Hardy-Weinberg equilibrium identified 37 outlier SNPs, which were removed using VCFtools. The total number of variable SNPs was 36,121, which reduced to 35,886 SNPs when only one SNP per locus was randomly retained.

### Population genetics and isolation by distance

The H_e_ and F_IS_ values ranged from 0.02 to 0.09 and 0 to 0.03, respectively (Fig. S1). The F_ST_ values between sampling sites ranged from 0.02 to 0.42 with comparisons to the geographically most distant and isolated population on Marie Galante (MG) having the highest values (ranging from 0.13–0.42 in all pairwise comparisons with an average of 0.25) (Fig. S2; Table S1). Excluding the MG site, the highest F_ST_ values (ranging from 0.05–0.13, with an average of 0.10) occurred in pairwise comparisons involving Ilets Pigeon (IP), another isolated, but near-shore, island population. Unsurprisingly, the F_ST_ values between IP and geographically proximate sites on the mainland of Basse Terre were the lowest of the pairwise comparisons involving IP (F_ST_ = 0.05 between IP and Ma, a nearby site on Basse Terre). The analysis of all Basse Terre and Grande Terre sites showed a significant pattern of isolation by distance (R^2^ = 0.059; *p* = 0.0001). The separate isolation by distance analysis of only Basse Terre samples was significant (R^2^ = 0.073; *p* = 0.0001), while the analysis of only Grande Terre samples was not significant (R^2^ = 0.0004; *p* = 0.419). The isolation by distance analysis of sites in the northern half of Basse Terre was not significant (R^2^ = 0.007; *p* = 0.273), but the analysis of southern sites was significant (R^2^ = 0.258; *p* < 0.0001). However, it should be noted that estimates of the Nm for all sites on Grande Terre and Basse Terre (where F_ST_ ranged from 0.02–0.18) exceed one (Nm = 2 - >20), which suggests high levels of gene flow and little opportunity for allelic fixation by drift despite a significant pattern of isolation by distance.

### Population structure

**Fig. 3:**
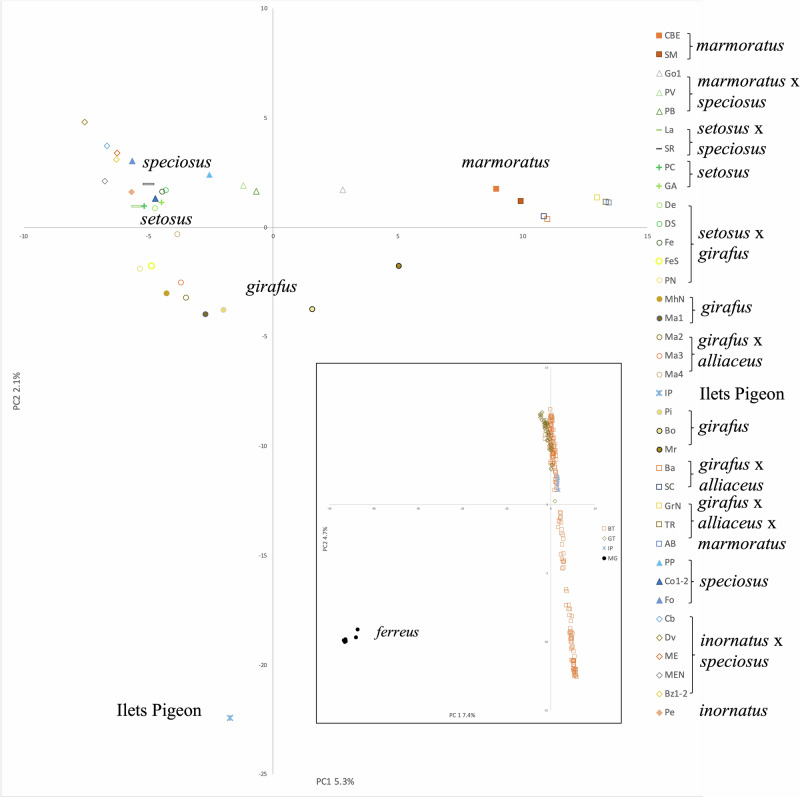
Non-spatial population structure. Average Principal Component Analysis (PCA) coordinates using the R package *adegenet* (Jombart 2008) of only *Anolis marmoratus* ssp. samples (*n* = 232) with 35,886 Single Nucleotide Polymorphism (SNP) markers. Inset shows PCA of all Guadeloupean anole (*Anolis marmoratus* ssp.) samples (*n* = 240) from Basse Terre (BT), Grande Terre (GT), Ilets Pigeon (IP), and Marie Galante with 36,059 SNP markers. Site abbreviations are listed in Table 1. Solid symbols represent subspecies sites according to Lazell (1964) and hollow symbols with the same shape are similar subspecies with varying degrees of intergradation.

Consistent with patterns of F_ST_ among sites, a PCA of SNP variation in all samples (*n* = 240) showed a major separation between the *A. ferreus* population on Marie Galante and all other *Anolis marmoratus* ssp. (Fig. 3 inset). When *A. ferreus* samples were excluded and the remaining *Anolis marmoratus* ssp. samples (*n* = 232) were analyzed, the Ilets Pigeon samples formed a distinct cluster in PC space (Fig. 3). The remaining samples from the five subspecies on Grande Terre and Basse Terre formed a continuum and the amount of variation explained by the first two principal coordinates was relatively low. The average PC coordinates of sites with the same subspecies tended to cluster together, but sites with multiple subspecies were clustered together (*A. m. inornatus*, *A. m. setosus*, *A. m. speciousus*) and sites with *A. m. girafus* were not all clustered together. Sites with samples from intermixed subspecies overlapped with subspecies and neighboring sites.

**Fig. 4:**
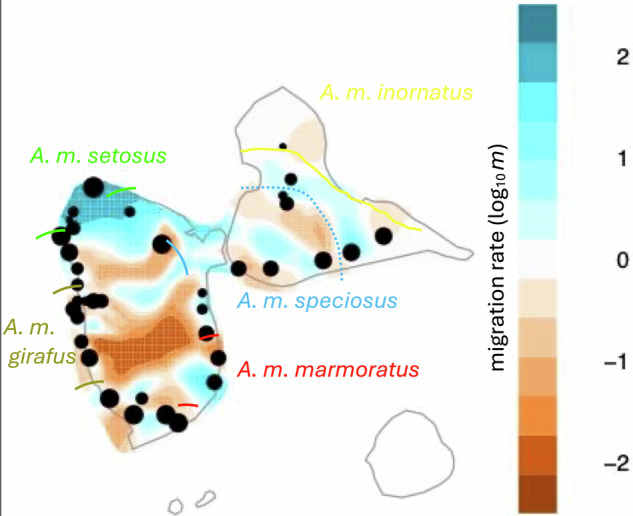
Spatially explicit map of effective migration rates. Estimated Effective Migration Surfaces (EEMS) posterior mean migration rates plot on the log_10_(m) scale (Petkova et al. 2016) of Guadeloupean anole (*Anolis marmoratus* ssp.) samples (*n* = 232) using 35,886 Single Nucleotide Polymorphism markers. The samples were from Basse Terre, Grande Terre, and Ilets Pigeon. Migration rates near 0 represent the expectation of isolation by distance, while higher values show areas of facilitated effective migration and negative values show areas of restricted effective migrtion. The size of the black circles corresponds to the sample size in a given area, which includes the grouping of some samples from closely located sites. The general location of the breaks between subspecies according to Lazell (1964) are designated with colored lines. The dashed blue line shows the general location of the escarpment transition between mesic and xeric habitat on Grande Terre.

The general visual pattern of gene flow the EEMS analyses created was similar for all the different deme sizes (results not shown). The EEMS analysis showed the strongest reduction in effective migration across the south-central region of Basse Terre, from east to west and extending to both coasts (Fig. 4). This region corresponds roughly to the area of highest elevation just north of the active La Souffriere volcano and spans a strong ecological gradient from lowland wet forest in the southeast of Basse Terre, to high elevation low forest of the central mountains, to the dry thorn scrub of the southwestern lowlands. Other weaker barriers to gene flow were detected in the northern quarter and southern sections of Basse Terre. In the north, the central mountain chain appears to restrict gene flow, but less so than in the higher elevation of the south. Additionally, a somewhat less restrictive corridor of gene flow was suggested that follows the Route de la Traversee, the main road that occupies a pass across the central mountain chain. Grande Terre did not show strong barriers to effective migration when compared to Basse Terre; however, reduced effective migration was associated with the habitat transition from the uplifted mesic southwest portion of the island to the xeric northern and eastern lowlands.

**Fig. 5:**
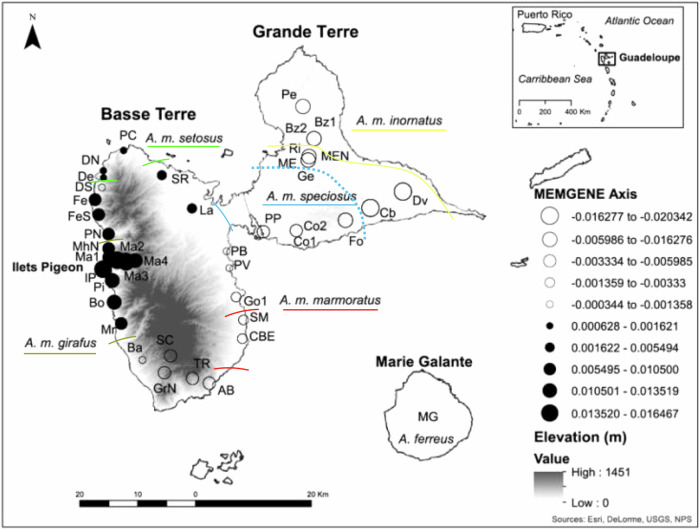
Spatially explicit map of fine-scale genetic variation. First MEMGENE axis produced by analyzing 232 samples from Basse Terre, Grande Terre, and Ilets Pigeon (Galpern et al. 2014). The individual genetic distance matrix used was estimated with the *bed2diffs_v1* script from the Estimate Effective Migration Surfaces analysis (Petkova et al. 2016). MEMGENE axis values are the positive and negative eigenvector values. Circles that are the same size and color are more similar to each other. The dashed blue line shows the approximate location of the escarpment transition between mesic and xeric habitat on Grande Terre. Site abbreviations are listed in Table 1.

The first four MEMGENE axes explained 45%, 14%, 9%, and 6% of the genetic variance, respectively. The adjusted R² value was 0.157, which reflects the amount of genetic variation that is explained by the spatial pattern in the data. The visual representation of spatial variation in MEMGENE uses black and white circles of different sizes to denote spatial clusters of genetically similar samples. The first major divide is between samples (individuals) that are either black or white. Then the circles that are the same color and similar size are the next most similar genetic cluster. The first MEMGENE axis showed groupings that in general match transition zones among subspecies (Fig. 5). Basse Terre was divided into two main groups with black circles in the west that became smaller in the northern section (approximate transition zone between and *A. m. girafus* and *A. m. setosus*) and white circles in the southeastern part of the island that became larger in the southern tip where *A. m. marmoratus* tranisition into the highly introgressed *A. m. girafus* x *alliaceus* x *marmoratus*. The pattern on Grande Terre was more homogenous with only white circles that had a slight shift in size across the transition zone between *A. m. inornatus* and *A. m. speciosus*. This zone on Grande Terre is where the environment transitions from the mesic southwest to the xeric northern and eastern portions of the island.

### Phenotypic analyses

**Fig. 6:**
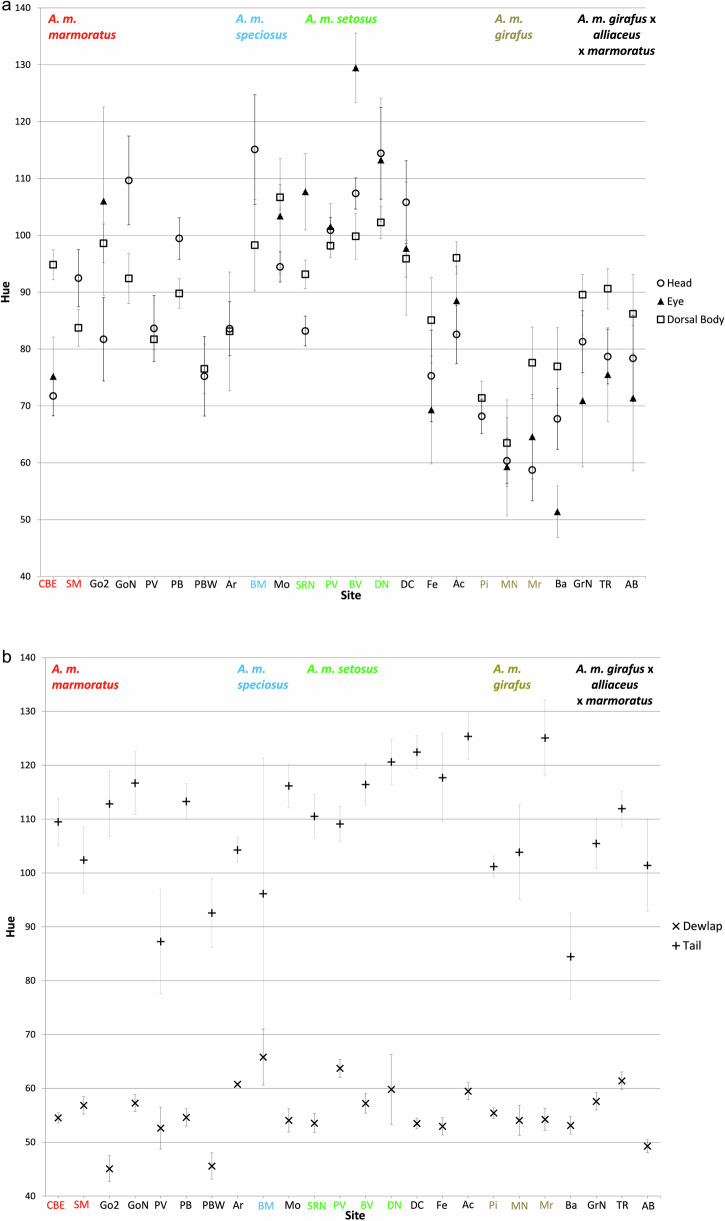
Spectral reflectance hue values of male lizard body parts. Average hue angle ± 1 SE for (**a**) dorsolateral head, eye, and dorsolateral body, and (**b**) dewlap and lateral tail parts of Guadeloupean anole (*Anolis marmoratus* ssp.) from Basse Terre. Locality abbreviations are described in Table 1. Subspecies intergrade as the sampling locality moves counter clockwise around Basse Terre. The sites that are the same color of the subspecies name are characteristic of that particular subspecies according to Lazell (1964). Average hue values of samples from Grande Terre are not shown and found in Muñoz et al. (2013).

The plots of hue angle for dorsolateral head, eye, and dorsolateral body all showed the same general pattern with a major decrease in values from sampling sites that have *A. m. setosus* to *A. m. girafus* (Fig. 6a). The plots of hue angle for each body part revealed that dewlap color and tail color do not vary substantially among sites (Fig. 6b). Dewlaps for all sites on the two main islands were consistently deep yellow in color and, while there was minor variation, it was not associated with subspecies. Tail color was more variable but tended towards a blue wash on a green background at all sites and, again, was not associated with subspecies.

We found a correlation between genetic distance (pairwise F_ST_/(1- F_ST_)) and phenotypic divergence in dorsolateral head and dorsolateral body color, but not in dewlap or lateral tail color (Fig. S3). The scatter plots of all samples for genetic distance versus dorsolateral head distance (R^2^ = 0.074; *p* = 0.0001) and dorsolateral body distance (R^2^ = 0.036; *p* = 0.007) were significant, while genetic distance versus dewlap distance (R^2^ = 0.011; *p* = 0.128) and tail distance (R^2^ = 0.016; *p* = 0.136) were not significant. The analysis of samples from each island separately also were significant for both dorsolateral head (Basse Terre R^2^ = 0.104; *p* = 0.009; Grande Terre R^2^ = 0.053; *p* = 0.026) and dorsolateral body color (Basse Terre R^2^ = 0.033; *p* = 0.039; Grande Terre R^2^ = 0.123; *p* = 0.022). However, we recognize that these R^2^ values are very low.

### Environmental impact on gene flow

**Fig. 7:**
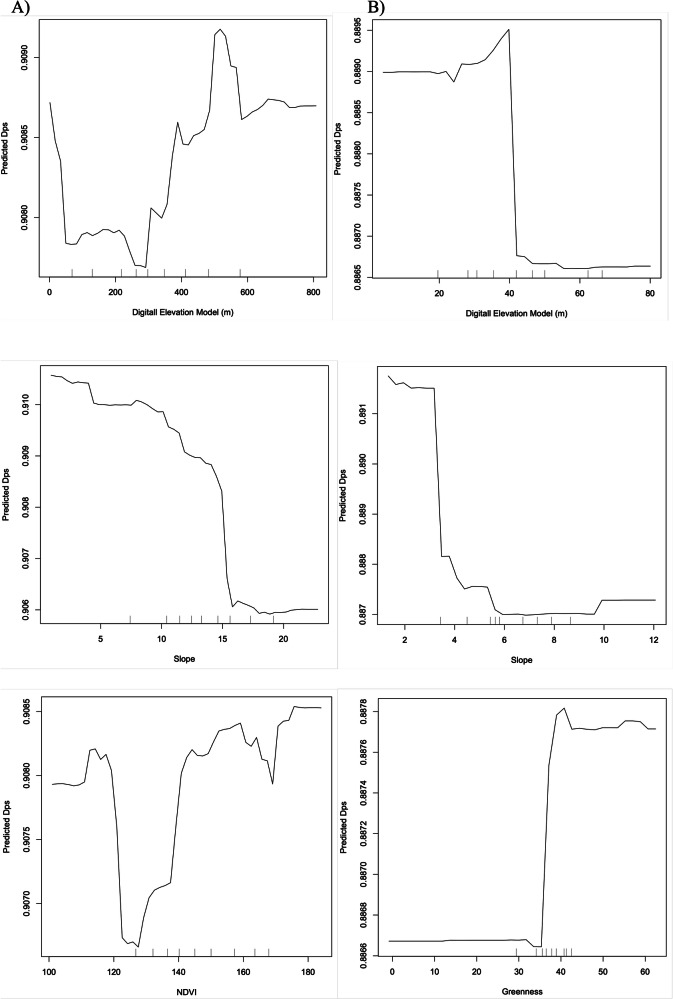
Influence of environmental variables on gene flow. Random Forest partial dependence plots created using the *partialDependence* program in R version 3.6.0 (R Core Team 2019) for (**A**) Basse Terre and (**B**) Grande Terre samples analyzed separately. The predicted proportion shared alleles (Dps) values are relative to an increase in the given environmental variable, which included elevation, slope, Normalized Difference Vegetation Index (NDVI), and Tassled Cap greenness (Kauth and Thomas 1976).

The Random Forest analyses were conducted to determine which environmental variables influenced Guadeloupean anole genetic differentiation. The optimized number of trees we used for the Random Forest analyses was 500. The Random Forest analysis of samples from Basse Terre explained 79% of the variance in genetic differentiation, which was substantially higher than the analysis of samples from Grande Terre (44%). Both islands had elevation and slope as top three variables for explaining genetic differentiation (Table S2). However, Basse Terre had NDVI (Normalized Difference Vegetation Index) while Grande Terre had greenness as a top three variable. The partial dependence plots for Basse Terre showed a non-linear relationship with predicted Dps (proportion of shared alleles) values for elevation and NDVI, while slope showed a decreasing relationship (Fig. 7). For Grande Terre, elevation and slope both showed a linear decreasing relationship, while greenness showed a steep increasing relationship with predicted Dps.

The GDM analysis models the influence of environmental, geographic, and phenotypic variables on Guadeloupean anole genetic differentiation. The percent deviance explained was 31% and six variables were identified as important. Dorsolateral body had the highest I-spline coefficient (0.019), followed closely by geographic distance (0.018) and dorsolateral head hue (0.016). The remaining variables included Bioclim 12 (I-spline coefficient = 0.007), slope (I-spline coefficient = 0.006), and elevation (I-spline coefficient = 0.005).

## Discussion

In this study, we used a landscape genetic approach to characterize the population genetic structure of Guadeloupean anoles and quantify the relative roles of the environment and isolation-by-distance (IBD) in reducing gene flow. We integrated genomic, environmental, and phenotypic data to test our hypotheses. Our first hypothesis was that subspecies on Basse Terre and Grande Terre would form distinct genetic clusters. Our Principal Component Analysis (PCA) showed a general separation of subspecies, but there was a lot of overlap in the placement of subspecies from type localities according to Lazell (1964). The most distinct clusters were formed by samples from Ilets Pigeon and Marie Galante, while the main island subspecies showed very low levels of genetic divergence and substantial gene flow among all sites. However, our spatially explicit Estimate Effective Migration Surfaces (EEMS) and MEMGENE analyses showed patterns that in general correspond to where subspecies diverge according to Lazell (1964), which demonstrates the role of the environment in the distribution of subspecies. Thus, we only found partial support for our first hypothesis.

Even though gene flow was generally high among sites, we found reduced gene flow among sites that was associated with differences in certain color phenotypes. The combined analyses of genetic differentiation and body coloration showed a positive correlation of increased genetic differentiation with increased disparity of dorsolateral head and dorsolateral body hue values. This provides support for isolation-by-adaptation in our study system. Other studies of anoles also have found correlations between genetic and morphological divergence. Ogden and Thorpe (2002) found that gene flow and morphological divergence was related to *A. roquet* from different habitats rather than from different phylogenetic lineages. In addition, Stenson et al. (2002) found genetic differentiation was high among morphologically different populations of the Dominica anole (*A. oculatus*) along a linear transect. In another lizard, wall lizard (*Teira dugesii*), Brown et al. (2023) found high rates of gene flow among animals from inland and beach sites, which had differences in their dorsal coloration). In their system, gene flow was stronger from inland to beach sites, which demonstrates an alternative way gene flow can be restricted.

We did not find a correlation between genetic differentiation and dewlap and lateral tail hue values. Dewlap coloration was the least variable of all phenotypic measures and was consistent across sites. Because dewlap coloration is important in species recognition (Losos 1985; Leal and Fleishman 2004; Nicholson et al. 2007), the similarity in color of the dewlap may be contributing to the continuation of gene flow among subspecies. Tail color also was fairly consistent in all sites and conspicuous tails may serve a general anti-predator function because the distal portion of the tail can be lost and regenerate (Fisher et al. 2012). In contrast, body coloration is likely adaptive with dull, cryptic colors favored in open habitats where predation from visual predators, such as kestrels, is more intense, and conspicuous colors favored in closed habitats where sexual selection for visible signals may be more important (Muñoz et al. 2013). We suspect maintenance of adaptive variation for different phenotypes is probably due to physical linkage of relatively small regions that have reduced rates of recombination. For example, Crawford et al. (2023) conducted a comparative genomic analysis of *A*. *m*. *marmoratus* and *A*. *m*. *speciosus* and found genomic islands of divergence between the two subspecies that relate to pigmentation genes. If Guadeloupean anole color phenotypes are controlled by a limited number of genes, then the color differences among subspecies can be maintained even though there are relatively high rates of neutral gene flow. This situation has been found in a non-lizard system where Mullen et al. (2009) found a role for a pigment gene (*Mc1r*) in the color differences of beach mice subspecies.

Our second hypothesis was that environmental variables would be more important than IBD at reducing gene flow and these differences would be greater on the more environmentally diverse Basse Terre than Grande Terre. Both our spatially explicit EEMS and MEMGENE analyses revealed a stronger pattern of spatial genetic structure on Basse Terre compared to Grande Terre with more departures from IBD and variable memgene axes among Guadeloupean anole sampling sites on Basse Terre. Interestingly, we were able to detect a subtle impact of the habitat gradient on gene flow on Grande Terre, with lower-than-expected gene flow (given the geographic distance) across the ecological gradient from the uplifted, mesic southwest to the low, xeric, northern and eastern lowlands. To test our second hypothesis we also needed to determine if IBD was present in our system. While there was a pattern of IBD in Basse Terre, we did not detect IBD in Grande Terre. We attribute the differences between islands to geographic scale, environmental variation, and Geological history. Linear geographic distances between the most distant sampling sites on Basse Terre were nearly 40 km and approximately 25 km on Grande Terre. When the maximum distances among sites was made more similar between the two islands, sites from the northern half of Basse Terre did not show a pattern of IBD, but sites from the southern part did show a pattern of IBD. We suspect that the pattern of IBD on the southern portion of Basse Terre may be driven more by isolation-by-environment due to the high environmental complexity in that part of the island. The southern Basse Terre region is characterized by the strongest ecological gradients in the archipelago and an active volcano, both of which may contribute to divergence of southern from northern Guadeloupean anoles due either to strong selection or a history of geographic isolation. Volcanic activity has decreased anole abundance on a different island (Muñoz and Hewlett 2011), which could increase divergence by restricting gene flow if a large area of habitat is severely altered by lava flow. In our Generalized Dissimilarity Modeling analysis, we found that phenotypic color differences were the most important variable in explaining reduced gene flow, but geographic distance was nearly equivalent. The phenotypic differences in Guadeloupean anoles correlate strongly with environmental differences. Thus, the environment and IBD were important in limiting gene flow. In a different species of Lesser Antilles anoles, Wang et al. (2013) showed geographic isolation explains more genetic divergence than ecological isolation. However, Wang et al. (2013) used a different analytical approach with structural equation modeling and did not include phenotypic data.

Our Random Forest analyses identified elevation and slope as important variables influencing genetic differentiation on both main islands. On each island, gene flow initially decreased as the values for elevation and slope increased. The habitat transition on Basse Terre from the coast to the mountainous interior is dramatic and can change from xeric, dry scrub habitat to rainforest. Thus, the different elevation and slope values are likely closely associated with differences in habitat. Elevation and slope also were important variables at explaining genetic differentiation on Grande Terre, but the range of environmental values was much smaller than on Basse Terre. Unexpectedly, the relationship between elevation and proportion of shared alleles (Dps) became the opposite with more gene flow at higher elevations on Basse Terre, but this could be an artifact of a low number of samples from high elevations. The Normalized Difference Vegetation Index values also were non-linear with initially restricted gene flow, but then facilitated gene flow, which was unexpected. On Grande Terre, gene flow increased dramatically with higher greenness values, which could reflect the sharp transition between mesic and xeric habitat on the island. As expected, the amount of genetic differentiation explained was much higher for the Basse Terre analyses compared to Grande Terre.

Our EEMS and MEMGENE analyses also both supported more restricted gene flow on Basse Terre compared to Grande Terre. The environmental gradients on Basse Terre were substantially more dramatic than the gradient on Grande Terre. We found a clear effect of environment on levels of gene flow among sites, with substantially less gene flow than expected across the southern portion of Basse Terre where the environmental gradient was the strongest, and at points along the rainfall gradients in the lowlands. We were not able to obtain lizard samples from the National Park, which encompasses the southern portion of the central mountain chain on Basse Terre, so sampling artifacts could be a concern. However, EEMS analysis is robust to sampling gaps in comparison to PCA and likely to recover the major patterns of gene flow (Petkova et al. 2016). Thus, our second hypothesis was partly supported.

### Speciation in *Anolis*

Isolation of Guadeloupean anoles on offshore islands has led to distinct genetic differentiation (i.e., Ilets Pigeon) and complete speciation in the case of *A*. *ferreus* on Marie Galante. The *A. ferreus* samples from Marie Galante showed the strongest genetic differentiation among all comparisons with other *A. marmoratus* samples and, together with their clear phenotypic divergence (Cope 1864; Underwood 1959), supports their recognition as a species. In a sense, the population of Marie Galante represents one end of the speciation spectrum represented by the Guadeloupean anoles. However, the *A. ferreus* samples from Marie Galante had an extremely low genetic diversity value compared to the other sites, which could have been due to a founder effect or bottleneck when the Marie Galante island was inhabited, and caused this site to have the highest genetic differentiation values. The samples from Ilets Pigeon showed moderate levels of genetic differentiation compared to other *A. marmoratus* ssp., but, excluding *A*. *ferreus*, they showed the strongest level of differentiation in our PCA. The population on Ilets Pigeon also may have been impacted by a founder effect (e.g., Kolbe et al. 2012), drift, or a bottleneck, but their level of genetic diversity and inbreeding was similar to most other sites. Ilets Pigeon anole samples did not group with *A*. *m*. *girafus* samples in our PCA, which does not support Lazell’s (1972) grouping of Ilets Pigeon anoles with *A*. *m*. *girafus*. While we did not analyze the phenotype of Ilets Pigeon samples, they are quite distinct in appearance from mainland Guadeloupean anoles and could be seen to represent an additional subspecies in this complex. Additional morphological analyses are needed to place the Ilets Pigeon population more clearly in the context of the speciation spectrum represented by the Guadeloupean anoles.

The Guadeloupean anole complex includes five additional subspecies on offshore islands (Lazell 1964). Genetic analyses on samples from these subspecies would shed additional light on the role of complete isolation in Guadeloupean anole genetic divergence. Our sampling also was mainly limited to coastal areas around Basse Terre. Additional sampling along elevational transects would provide a more direct investigation of the impact of elevation and slope on gene flow. Our study integrated genomic, phenotypic, and environmental data into a single analysis and is an approach that could be used in other systems to investigate the roles of geographic isolation and the environment in reducing gene flow. The integration of genomic, phenotypic, and environmental data into a single analysis is critical for investigating the roles of geographic isolation and the environment in affecting gene flow. This approach could be applied to other systems to elucidate general patterns.

## Supplementary information

Supplemental Figures and Tables

Supplemental Material - ddRADSeq

Supplemental Material - Grande Terre Genetic File for Random Forest Analyses

Supplemental Material - Basse Terre Genetic File for Random Forest Analyses

Supplemental Material - Generalized Dissimilarity Modeling Data

## Data Availability

The cleaned fastq files are deposited in NCBI’s SRA database under BioProject accession number PRJNA1145996. Our genotype and phenotype data used for our Random Forest and Generalized Dissimilarity Modeling also are available in the supplementary material.
