# Supplementary material for: Influence of geographic isolation and the environment on gene flow among phenotypically diverse lizards
Source: Heredity (Edinb). 2024 Sep 12;133(5):317–30. doi: 10.1038/s41437-024-00716-y (PMC11528109; doi:10.1038/s41437-024-00716-y)
Supplement: Supplementary file 1 — Supplemental Figures and Tables [file 41437_2024_716_MOESM1_ESM.docx]

Figure S1. Expected heterozygosity (H_e_) and inbreeding coefficient (F_IS_) ± standard error estimated using the populations program in Stacks version 2.41 (Catchen et al. 2011, 2013). Site abbreviations are the same as in Table 1.

Figure S2. Color plot of pairwise F_ST_ values estimated using the *populations* script in Stacks versions 2.41 (Catchen et al. 2011, 2013). Site abbreviations are the same as in Table 1.

A.

Table S1. Pairwise F_ST_ values estimated using the *populations* script in Stacks versions 2.41 (Catchen et al. 2011, 2013). Site abbreviations are the same as in Table 1.

Figure S3. Mantel tests between the F_ST_/(1- F_ST_) (Rousset 1997) and log base 10 of hue values using GenAlEx version 6.5 (Peakall and Smouse 2006, 2012) with 9,999 permutations and an alpha level of 0.05 to determine the significance of the simulated p-values. The analyses were conducted on samples from 24 sites, which also included eight sites from Grande Terre that are reported in Muñoz et al. (2013).

A)

B)

C)

D)

Table S2. Random Forest average percent increase in mean squared error (IncMSE) and node purity (IncNP) with standard deviation (SD) for Basse Terre (BT) and Grande Terre (GrTr) analyzed separately. The geographic variables included slope, elevation, and aspect while the biologic variables included Normalized Difference Vegetation Index (NDVI) and Tassled Cap brightness, greenness, and wetness (Kauth and Thomas 1976).

| **Variables** | **BT IncMSE (SD)** | **BT IncNP (SD)** | **GrTr IncMSE (SD)** | **GrTr IncNP (SD)** |
| --- | --- | --- | --- | --- |
| Slope | **22.774 (1.239)** | **0.0179 (0.0003)** | **9.872 (0.892)** | **0.00157 (0.00006)** |
| Elevation | 18.712 (1.835) | 0.0126 (0.0002) | 6.025 (1.29) | 0.00145 (0.00004) |
| Aspect | 18.512 (0.821) | 0.0140 (0.0002) | 3.161 (1.44) | 0.00080 (0.00007) |
| NDVI | 15.023 (1.474) | 0.010 (0.0002) | -0.058 (1.59) | **0.00249 (0.00004)** |
| Tasseled Cap Brightness | 11.354 (1.094) | 0.0091 (0.0003) | 0.178 (0.465) | 0.00076 (0.00002) |
| Tasseled Cap Greenness | 15.162 (0.603) | 0.0091 (0.0003) | 2.669 (1.20) | 0.00111 (0.00005) |
| Tasseled Cap Wetness | 14.556 (0.436) | 0.0122 (0.0001) | 0.004 (0.624) | 0.00074 (0.00007) |
