# Supplementary material for: Influence of geographic isolation and the environment on gene flow among phenotypically diverse lizards
Source: Heredity (Edinb). 2024 Sep 12;133(5):317–30. doi: 10.1038/s41437-024-00716-y (PMC11528109; doi:10.1038/s41437-024-00716-y)
Supplement: Supplementary file 2 — Supplemental Material - ddRADSeq [file 41437_2024_716_MOESM2_ESM.docx]

Appendix S1

Extracted genomic DNA was quantified using the Invitrogen Qubit dsDNA HS Kit (ThermoFisher Scientific, Waltham, MA). Each sample was standardized to 500 ng of DNA and digested with SphI-HF® (New England Biolabs, Ipswich, MA) and MluCI® (New England Biolabs, Ipswich, MA) according to manufacturer’s instructions. We used either Agencourt® AMPure XP magnetic beads (Beckman Coulter Genomics, Danvers, MA) or home-made Sera-Mag SpeedBeads (GE Healthcare, Pittsburgh, PA) magnetic beads (Rohland and Reich, 2012) for all purification steps. Digested and cleaned DNA samples were quantified using the Qubit High Sensitivity DNA Kit and all sets of samples were standardized to either 120 ng or 150 ng for the adapter ligation step. Samples were labeled with 12 adapters and purified using magnetic beads. Adapter ligated samples were quantified using the Qubit High Sensitivity DNA Kit and all samples were standardized to the same quantity. Sets of 12 samples with unique adapters were pooled and size selected for 202 base pairs (bp) with a range of 182 bp to 222 bp using 2% ethidium free cassettes on the Pippin Prep (Sage Science, Beverly, MA). Sets of four pools that each contained 12 samples were polymerase chain reaction (PCR) amplified to increase the quantity of each pool and incorporate a unique index. The KAPA HiFi™ Real-Time PCR Library Amplification Kit (Kapa Biosystems, Wilmington, MA) was used according to manufacturer’s instructions for 12 PCR cycles. Index labeled pools were purified and quantified using the Agilent High Sensitivity DNA kit (Agilent Technologies, Santa Clara, CA) according to manufacturer’s instructions. Equimolar amounts of each of the four pools were combined and purified. The final pool was quantified again using the Agilent High Sensitivity DNA kit and the library was submitted to the Northwest Laboratory Bauer Core Facility at Harvard University for single-end 50 bp read sequencing using an Illumina HiSeq™ 2000 sequencing system (San Diego, CA). Six sets of libraries that each included 48 samples were created. A total of eleven next-generation sequencing runs were conducted. Four libraries were run one time, one library was run two times, and one library was run five times, which included one paired-end run.

The Stacks *process_radtags* program was run to clean the data, discard reads with low quality scores, and rescue barcodes and RAD-Tags. Repeated sample files were merged after the *process_radtags* step. The assembly parameters for the *de novo* processing of the samples were optimized by analyzing a subset of samples, n = 2 per site, and varying the parameters *M* (“number of mismatches allowed between the two alleles of a heterozygote sample) and *n* (“number of mismatches allowed between any two alleles of the population”) to optimize the number of SNP loci (Rochette and Catchen, 2017). The Stacks *populations* script was run with the parameters: minimum number of populations a SNP has to be present in (-p 28), minimum percentage of individuals that have a SNP (-R 0.8), minimum minor allele frequency (--min-maf 0.01), minimum minor allele count (--min-mac 3), and maximum observed heterozygosity (--max-obs-het 0.7). The program VCFtools version 0.1.16 (Danecek et al., 2011) was used to remove samples that had > 50 % missing data.
